# Non-Traditional Antibacterial Screening Approaches for the Identification of Novel Inhibitors of the Glyoxylate Shunt in Gram-Negative Pathogens

**DOI:** 10.1371/journal.pone.0051732

**Published:** 2012-12-11

**Authors:** Kelly C. Fahnoe, Mark E. Flanagan, Glenn Gibson, Veerabahu Shanmugasundaram, Ye Che, Andrew P. Tomaras

**Affiliations:** Antibacterials Research Unit, Pfizer Worldwide Research & Development, Groton, Connecticut, United States of America; The Scripps Research Institute and Sorrento Therapeutics, Inc., United States of America

## Abstract

Antibacterial compounds that affect bacterial viability have traditionally been identified, confirmed, and characterized in standard laboratory media. The historical success of identifying new antibiotics via this route has justifiably established a traditional means of screening for new antimicrobials. The emergence of multi-drug-resistant (MDR) bacterial pathogens has expedited the need for new antibiotics, though many in the industry have questioned the source(s) of these new compounds. As many pharmaceutical companies' chemical libraries have been exhaustively screened via the traditional route, we have concluded that all compounds with any antibacterial potential have been identified. While new compound libraries and platforms are being pursued, it also seems prudent to screen the libraries we currently have in hand using alternative screening approaches. One strategy involves screening under conditions that better reflect the environment pathogens experience during an infection, and identifying *in vivo* essential targets and pathways that are dispensable for growth in standard laboratory media *in vitro*. Here we describe a novel screening strategy for identifying compounds that inhibit the glyoxylate shunt in *Pseudomonas aeruginosa*, a pathway that is required for bacterial survival in the pulmonary environment. We demonstrate that these compounds, which were not previously identified using traditional screening approaches, have broad-spectrum antibacterial activity when they are tested under *in vivo*-relevant conditions. We also show that these compounds have potent activity on both enzymes that comprise the glyoxylate shunt, a feature that was supported by computational homology modeling. By dual-targeting both enzymes in this pathway, we would expect to see a reduced propensity for resistance development to these compounds. Taken together, these data suggest that understanding the *in vivo* environment that bacterial pathogens must tolerate, and adjusting the antibacterial screening paradigm to reflect those conditions, could identify novel antibiotics for the treatment of serious MDR pathogens.

## Introduction

The threat of multi-drug-resistance in Gram-negative pathogens has grown from being a rare, isolated incident to an inevitability occurring worldwide. Antibiotic use and misuse has provided the evolutionary pressure necessary for the emergence and spread of new antibiotic-resistance mechanisms which, when combined with pre-existing mechanisms, have the potential to synergize and further complicate an already exhausted therapeutic arsenal for clinicians [1], [2]. Pathogens have become so recalcitrant to conventional antimicrobial therapy that combinations of antibiotics, or antibiotics with known toxicity liabilities are required in order to have a chance at defeating them. In many cases these approaches prove unsuccessful, leading to extended hospitalizations, and can result in costly device replacements, life-altering amputations, or even death. Organisms which have consistently made their way to the top of the public health threat list worldwide include *P. aeruginosa*, *Acinetobacter baumannii*, *Klebsiella pneumoniae*, and the extended-spectrum β-lactamase (ESBL)-producing members of the *Enterobacteriaceae* [3]. Of particular concern to the infectious diseases community has been the identification of new sources of antibacterial compounds for testing against these serious MDR pathogens [4]. And while much of this concern is warranted due to the underwhelming number of leads recently identified from both target-based and traditional whole cell screening efforts [5], the development and implementation of novel, non-traditional whole cell screens – using the same compound libraries already in existence – have yet to be described.

Targeting virulence to mitigate the infectivity of Gram-negative pathogens is not a new concept in antibacterial drug development. Ideas about preventing bacterial adherence to host cells [6], neutralizing toxins [7], disrupting biofilms [8], [9], or interfering with quorum sensing [10], [11] are all worthwhile approaches that have each demonstrated some promise in serving as weapons in the war against infectious diseases. Another approach to targeting virulence is to identify bacterial pathways that are required for cells to survive the harsh, nutrient-limited environment to which they must adapt in an infection, and this is a research area that has recently been explored in *A. baumannii* [12]. Certainly the physiological state of pathogens is dramatically different when they are residing within a host relative to when they are growing *in vitro* in a nutrient-replete medium, therefore it is critical to identify screening strategies that more closely mimic the environment these bacteria encounter during an infection. In the case of pathogens that colonize and persist in the pulmonary environment, such as *P. aeruginosa*, the bacteria are presented with a unique set of physiological challenges that they must tolerate in order to survive. Among these challenges is the metabolic requirement to utilize phosphatidylcholine (PC), which is the major component of lung surfactant and serves as the primary source of carbon in the lung. As lung surfactant is required for pulmonary function, PC is always present in the lung and is available to whatever pathogens choose to inhabit that organ. In the case of *P. aeruginosa*, it has been shown that this versatile Gram-negative pathogen preferentially migrates towards this phospholipid [13], having all of the tools necessary to disassemble and transport PC into the cell. After bacterial lipases liberate the long-chain fatty acids from PC, fatty acid transporters in the outer membrane are responsible for importing these lipids into the cell [14]. Once in the cytoplasm, these long chain fatty acids are iteratively reduced by β-oxidation, providing a free acetyl-CoA from each round of this cycle. These two carbon compounds are then shuttled into the TCA cycle, where they are then processed by the bypass pathway known as the glyoxylate shunt [15].

The glyoxylate shunt is composed only of two enzymes, isocitrate lyase (ICL) and malate synthase (MS), and is fairly well conserved amongst several bacterial species (72- and 65%-amino acid identity for ICL and MS, respectively, across *P. aeruginosa*, *A. baumannii*, and *Burkholderia cepacia*). Importantly, no human ortholog of this pathway has been identified, potentially making it a suitable target for antibacterial therapy. The existence of this TCA bypass is to conserve carbons that are normally expended, in the form of CO_2_, during the normal progression of the TCA cycle. These conserved carbons serve as substrates for gluconeogenesis, where they are ultimately incorporated into new molecules of glucose. In an environment where carbon is not available in an ample supply, the conservation of metabolized carbon is crucial for cell survival, hence the existence and function of the glyoxylate shunt. Because of the essential nature of this pathway when lipids are provided as the sole carbon source, we sought to exploit this phenotype by screening compounds for antibacterial activity in a defined, minimal medium. Our efforts demonstrate that alternative screening approaches can identify new lead material from existing compound collections that have previously shown no antibacterial activity when screened under traditional conditions.

## Methods

### Ethics statement

All animal procedures performed were in accordance with regulations and established guidelines and were reviewed and approved by the Pfizer Institutional Animal Care and Use Committee.

### Bacterial strains and media used

*P. aeruginosa* PAO1, kindly provided by Mike Vasil (University of Colorado at Denver), was used to construct deletion mutants and for the high-throughput screen. *E.coli* DH5α was used as a host strain for all genetic manipulations, and 1545-08 is a CTX-M-15-β-lactamase expressing clinical isolate. *K. pneumoniae* strain MGH78578 is a clinical isolate that has been described previously [16]. *A. baumannii* strain AB-3167 is a recent clinical isolate that was provided by International Health Management Associates, Inc (Schaumburg, IL). All strains were routinely cultured in Luria Bertani (LB) broth and agar. For determination of glyoxylate shunt essentiality for growth when fatty acids are provided as the sole carbon source, M9 minimal medium (Difco) was used, with 0.5% potassium acetate included as the sole carbon source in place of glucose. This medium, hereafter referred to as M9 Acetate, was used for high-throughput screening. Minimum inhibitory concentration (MIC) assays were conducted in both Mueller Hinton Broth (MHB) and M9 Acetate media in accordance with Clinical and Laboratory Standards Institute (CLSI) guidelines [17].

### Construction of *P. aeruginosa* glyoxylate shunt mutants

Site-directed deletion mutants of the ICL (encoded by *aceA*/PA2634) and MS (encoded by *glcB*/PA0482) genes were generated using allelic exchange with the *P. aeruginosa* suicide vector pEX100T [18]. Briefly, genes were PCR amplified with the following primer sets: *aceA* forward (5′-CTGTTCGTCTCCACCCC-3′) and *aceA* reverse (5′-GCCCCTGTTCCTTAGTGG-3′); *glcB* forward (5′-ATCCAGTCGTAACGCGAC-3′) and *glcB* reverse (5′-TTTCGTTGCGCTGCGGCG-3′) and the resulting amplicons were cloned into pCR2.1-TOPO (Invitrogen). Internal *Eco*NI and *Stu*I restriction sites were used to remove 995- and 1083-bp internal regions of the *aceA* and *glcB* genes, respectively. The Flippase Recognition Target (FRT)-containing gentamicin-resistance (Gm^R^) cassette was excised from pPS856 [19] by *Sma*I digestion, and ligated into these clones with T4 DNA Ligase (New England Biolabs) to replace the removed internal fragments, and the resulting plasmids were transformed into *E. coli* Top10 (Invitrogen). Plasmid DNA from Gm^R^ transformants was confirmed by DNA sequencing, digested with *Pvu*II and *Sca*I, and ligated into *Sma*I-digested pEX100T. After transformation into *E. coli* Top10, Gm^R^ transformants were confirmed by restriction digestion and used to deliver these deletion constructs into *P. aeruginosa* PAO1 via tri-parental mating using the helper plasmid pRK2013 [20]. Double-crossover recombination events were selected by first plating exconjugants on *Pseudomonas* Isolation Agar (PIA, Difco) containing 75 μg ml^−1^ Gm and incubating overnight at 37°C. Multiple colonies were collected and struck for isolation on LB agar containing 75 µg ml^−1^ Gm and 5% sucrose, to counterselect for the *sacB* marker on pEX100T. Gm^R^ Sucrose^R^ colonies were recovered and confirmed to be free of pEX100T by plating on LB agar containing 500 µg ml^−1^ carbenicillin. The integrity of strains that were sensitive to carbenicillin was further confirmed by PCR and DNA sequencing using *aceA*- or *glcB*-specific primers. To construct the double *aceA glcB* deletion mutant, we first excised the Gm^R^ marker from Δ*aceA* using pFLP2 [19], and then introduced the pEX100T/*glcB*:: Gm^R^ construct into this unmarked mutant using the same methods described above.

### *In vitro* and *in vivo* assessment of glyoxylate shunt mutants

To demonstrate the roles of ICL and MS in the ability of *P. aeruginosa* to utilize different sole carbon sources, wild-type PAO1, the Δ*aceA* and Δ*glcB* single mutants, and the Δ*aceA* Δ*glcB* double mutant were grown in both LB broth and M9 medium supplemented with 0.5% glucose, acetate, or succinate overnight at 37°C with shaking at 265 rpm. Viability differences were quantified by measuring the optical density at 600 nm (OD_600_) using spectrophotometry. The virulence of PAO1 and its isogenic glyoxylate shunt mutants was assessed using an alginate-based murine pulmonary model of infection. Briefly, alginate was extracted from a hyper-alginate-producing cystic fibrosis of *P. aeruginosa* as described previously [21]. 10 CF-1 mice (Charles River Laboratories) per group were made neutropenic by administering cyclophosphamide orally both 4 days and 1 day prior to bacterial challenge using 150 and 100 mg kg^−1^ doses (in 10 ml kg^−1^ sterile water), respectively. On the day of infection, overnight LB-grown cultures of each *P. aeruginosa* strain were collected by centrifugation, washed and serially diluted in phosphate buffered saline, and the 10^−7^ dilution was suspended in 11 mg ml^−1^ purified alginate. 50 μl of this suspension (∼500 CFU) was inoculated intranasally into mice that were anesthesized with isofluorane. Mice were sacrificed at both 2 and 48 hours post-infection, at which time lungs from each animal were aseptically harvested, homogenized, diluted, and plated to quantify *P. aeruginosa* levels. Murine septicemia challenges were also conducted with CF-1 mice (n = 5 per group), and overnight LB-grown *P. aeruginosa* cultures were normalized to an OD_600_ of 1.0, followed by serial dilution in 10-fold increments to 10^−8^. 500 μl of the 10^−5^, 10^−6^, 10^−7^, and 10^−8^ dilutions were administered via intraperitonial injection, and mice were monitored daily for early signs of mortality and/or death. All animal procedures performed were in accordance with regulations and established guidelines and were reviewed and approved by an Institutional Animal Care and Use Committee.

### Development of a high-throughput Screen (HTS) to identify glyoxylate shunt inhibitors in *P. aeruginosa*

HTS of 149,624 compounds, screened at final concentrations of 80 µM, was conducted in M9 Acetate with *P. aeruginosa* PAO1 in a 384-well microtiter plate format. For internal control purposes, each plate contained 12 wells of untreated PAO1 and 12 wells of PAO1 treated with 3-nitropropionate, a known ICL inhibitor described previously [22]. An overnight culture of PAO1 was grown in LB broth at 37°C with shaking, and the resulting cells were washed with M9 and adjusted to an OD_600_ of 0.5. This suspension was diluted 1∶100 into fresh M9 Acetate medium, and 50 µl was dispensed into the compound-containing wells of 384-well microtiter plates. Plates were incubated at 37°C for 24 h in a humidified incubator to avoid evaporation. Growth inhibition effects were quantified by measuring the OD_600_ and comparing those values to the internal controls included on each plate, and Z' and % CV values [23] were calculated from 5 representative 384-well plates. Based on these plates, the assay was calculated to have a Z' of 0.93 and a %CV of 3.33%, which confirmed the robustness and reproducibility of this assay.

### Purification of *P. aeruginosa* ICL and MS

To evaluate the specific inhibitory effects of compounds identified in the HTS, we cloned, overexpressed, and purified the two enzymes which comprise the glyoxylate shunt in *P. aeruginosa*. To do so, we first PCR amplified the full-length *aceA* and *glcB* genes from *P. aeruginosa* PAO1 using the following primers: *aceA* OE forward (5′-CATATGTCCGCATATCAGAACGAG-3′) and *aceA* OE reverse (5′-GGATCCTTAGTGGAACTGGTTCATGG-3′); *glcB* OE forward (5′-CATATGACTGAACGCGTTCAAGTC-3′) and *glcB* OE reverse (5′-GGATCCCTACAGCCCGTTCTTCGC-3′). Primers were designed to engineer *Nde*I and *Bam*HI restriction sites (underlined) at the 5′ and 3′ ends of the amplicons, respectively. Products were cloned into pCR2.1-TOPO (Invitrogen), verified by DNA sequencing, and subcloned into the cognate sites of the N-terminal 6x-His plasmid pET15b (Novagen). Integrity of the resulting constructs was verified by restriction digestion, and plasmids were transformed into *E. coli* BL21. An ampicillin-resistant transformant of each construct was used to inoculate 4 L of Terrific Broth (Difco), and cultures were incubated at 37°C shaking (300 rpm) until an OD_600_ of 0.5 was reached. Induction of the T7 polymerase was achieved by adding 0.5 mM isopropyl β-D-1-thiogalactopyranoside (IPTG), and cultures were incubated for another 4 h. Cells were harvested by centrifugation, washed once in Dulbecco's Phosphate Buffered Saline and suspended in a minimal volume of the appropriate lysis buffer (ICL, 50 mM potassium phosphate pH 7.5, 300 mM NaCl, 10 mM imidazole, 1 mM TCEP, 5% glycerol; MS, 20 mM Tris pH 7.5, 100 mM NaCl, 5 mM imidazole). Complete EDTA-free protease inhibitor cocktail (Roche) tablets were added to each lysis buffer just prior to conducting two rounds of mechanical lysis using a microfluidizer. Cell debris was removed by centrifugation for 30 minutes at 30,000×g at 4°C. Clarified ICL lysate was loaded onto a His-Select High Flow Nickel Affinity resin (Sigma) equilibrated in the above defined ICL buffer. The column was washed with this buffer until the OD_280_ returned to baseline, followed by elution with a 10-column-volume linear gradient (250 ml) of ICL buffer to a final concentration of 250 mM imidazole. Fractions containing ICL were pooled, concentrated, and applied to an S300 26/60 gel filtration column (GE Healthcare) pre-equilibrated in 50 mM potassium phosphate pH 7.5, 300 mM NaCl, 1 mM TCEP, 5% glycerol for further polishing and buffer exchange. Clarified MS lysate was purified as described above for ICL except that MS buffer (defined above) was used instead of ICL buffer, and that 25 mM imidazole was used for elution instead of 250 mM. Imidazole was removed from the MS prep by dialysis prior to concentration and addition of 5% glycerol for stability.

### ICL and MS IC_50_ determinations

ICL and MS assays were conducted based on methods described previously [24], [25], with modifications made to enable high throughput screening of compound libraries. ICL activity was determined in 50 µl reactions in a 384-well clear microtiter plate (Costar 3702) at 324 nm in a SpectraMax Plus 384 (Molecular Devices) pre-equilibrated to 30°C. Inhibitors solvated in DMSO were spotted to the assay plates using a Biomek FX (Beckman Coulter) and incubated for 5 minutes with enzyme buffer containing final concentrations of 30 mM imidazole pH 6.8, 5 mM MgCl_2_, 1 mM EDTA, 4 mM phenylhydrazine, and 0.5 pmoles ICL protein. Reactions were initiated with 250 µM DL-isocitric acid. MS activity was determined by measuring the amount of free coenzyme-A liberated when glyoxylate and acetyl-coenzyme A were catalytically condensed to malate and coenzyme-A. This reaction was monitored kinetically at 412 nm in the presence of a colorimetric substrate, 5,5′-dithio(2-nitro-benzoic acid) (DTNB), which has a strong absorbance at this wavelength upon cleavage by a thiol group (from coenzyme A-SH) and ionization at neutral pH. Enzyme buffer containing 50 mM potassium phosphate pH 7.5, 10 mM MgCl_2_, 0.2 mM acetyl-CoA, and 0.5 pmoles MS protein was pre-incubated with inhibitors for 5 minutes prior to initiating the reaction with buffer containing 50 mM potassium phosphate pH 7.5, 10 mM MgCl_2_, 0.5 mM glyoxylic acid (pH adjusted to 7.5 with 10 N sodium hydroxide), and 0.2 mM DTNB. Inhibitor potency was assessed based on the percent effect of both fully inhibited controls for each enzyme (ICL, 3-nitropropionate; MS, no enzyme) as well as uninhibited controls. The Z' and % CV values [23] were carefully monitored to ensure each assay was meeting our robust and well defined assay parameters.

### Homology modeling and induced fit docking

Homology models for *P. aeruginosa* ICL and MS were built using *M. tuberculosis* ICL [26] and MS [27] crystal structures as templates, respectively. The sequence alignment to the template structures was conducted using the Pfam [28] protein families database. Homology models were built with MODELLER 9v7 [29] and the loops were refined using the refine loops tools with the extended high-loop refinement procedure. Homology models were inspected to ensure that the side chains of the conserved residues were aligned to the template. Profile-3D [30] was used to assess the quality of the models as well as the protein report tool. The side chain positions of the ligand binding site residues in each model were refined by docking HTS hits into the site using induced fit docking from Schrodinger [31], which allows receptor flexibility. The final models were selected after multiple iterations of model construction and refinement.

## Results and Discussion

### *In vitro* and *in vivo* survival of *P. aeruginosa* requires a functional glyoxylate shunt

**Figure 1:**
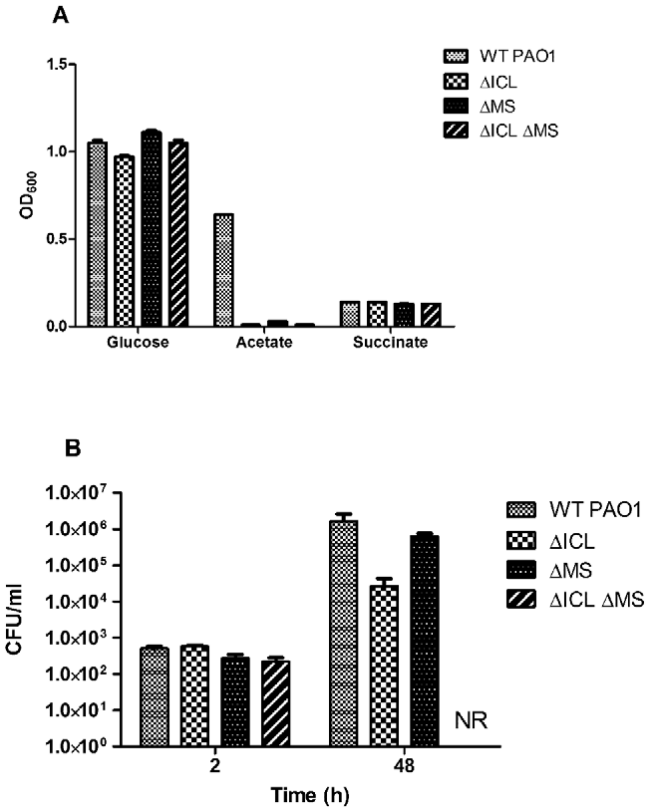
*P. aeruginosa* glyoxylate shunt mutants are deficient for growth both *in vitro* and *in vivo.* (A) The ability of wild-type *P. aeruginosa* PAO1 and its isogenic glyoxylate shunt mutants to utilize various sole carbon sources was assessed spectrophotometrically after overnight growth at 37°C. (B) The ability of these strains to colonize and persist in a murine lung model of infection was measured at 2- and 48-hours post-infection by lung homogenization and subsequent CFU ml^−1^ determination. NR – no recoverable colonies.

The idea of targeting the glyoxylate shunt for antimicrobial therapy has been described previously [32]–[34]. While the most significant consideration and progress in identifying such an inhibitor has been conducted with *M. tuberculosis* [35], the orthologous pathway in *P. aeruginosa* has also been implicated numerous times in various virulence and *in vivo* survival models [36], [37]. Interestingly, these have included both mammalian and non-mammalian hosts. To confirm these previous findings, we first constructed deletion mutants of the individual ICL- and MS-encoding genes (*aceA* and *glcB*, respectively), as well as a double knockout to see if any additive effects may exist. As expected, all three mutants were completely unable to grow on M9 Acetate, whereas no growth defects were witnessed, relative to the PAO1 parent strain, when either glucose or succinate was provided as the sole carbon source (Figure 1A).

Each of these mutants, along with the wild-type strain, was then used to infect mice in both septicemia and pulmonary models of infection. Given that the glyoxylate shunt has been shown to be essential for survival in environments where lipids are the primary source of carbon (such as in lung surfactant), it was not surprising that no differences in virulence were seen when these strains were tested in a septicemia model of infection (data not shown). When the virulence potential of these strains was tested in a pulmonary model of infection, however, we discovered that recovery of the single *aceA* mutant was significantly reduced, relative to wild-type, at 48 hours post-infection (Figure 1B). This finding did not seem to be a consequence of initial *in vivo* survival defects of the *aceA* mutant, however, as bacterial burdens of all strains tested were equivalent at 2 hours post-infection. The ∼50-fold decrease in virulence of an ICL-deficient strain of *P. aeruginosa* is consistent with data previously shown in other infection models [36]. Contrary to the *aceA* mutant, however, the *glcB* mutant did not show any attenuation in this model, with similar bacterial recovery seen between this strain and the wild-type PAO1 (Figure 1B). To our knowledge, this is the first report of an MS-deficient strain of *P. aeruginosa* being tested in a pulmonary model of infection, although such mutants have been shown to be involved in virulence in an alfalfa seedling model [38]. We have speculated that the ICL-mediated production of succinate from isocitrate is sufficient to promote *in vivo* survival, despite the accumulation of glyoxylate. Perhaps most interestingly, we found the double *aceA glcB* mutant to be severely attenuated in this model, much more so than the single *aceA* mutant, with no recoverable colonies at 48 hours despite equal lung burdens at 2 hours (Figure 1B). These data suggest that no bypass mechanisms can circumvent the loss of both enzymes in the glyoxylate shunt. Taken together, these *in vivo* results demonstrate that glyoxylate shunt inhibitors that have the greatest potential to serve as therapeutic agents against *P. aeruginosa* need to inhibit both enzymes in the pathway. One potential caveat to this approach, however, is the likelihood that glyoxylate shunt inhibitors would only be efficacious in the pulmonary environment, as evidenced by the lack of virulence differentiation between wild-type PAO1 and its isogenic glyoxylate shunt mutants when mice were challenged in a septicemia model of infection. Additionally, the impact on pulmonary efficacy when non-lipid-based carbon sources are introduced (namely glucose) needs to be explored and assessed when considering this target for antimicrobial therapy.

### High-throughput screening identifies compounds that inhibit *P. aeruginosa* growth in M9 acetate

Given the clear differences in growth *in vitro* between wild-type PAO1 and its isogenic glyoxylate shunt mutants, we were intrigued by the possibility that this metabolic pathway could represent a new antibacterial target. Accordingly, we utilized M9 Acetate to screen for compounds that affected growth of wild-type PAO1. We set our cutoff at 40% growth inhibition relative to the untreated controls in each plate, and the screening of approximately 150,000 compounds yielded 498 primary hits, resulting in a 0.3% hit rate. 219 of the primary hits were subjected to a secondary screen that consisted of repeat growth inhibition in M9 Acetate, as well as an assessment of growth inhibition, if any, in M9 Glucose. The latter was included to triage compounds that had intrinsic whole cell activity that was not associated with the functionality of the glyoxylate shunt. Additionally, this assay also served to eliminate any non-specific, detergent-like compounds that had whole cell activity due to membrane disruption rather than a defined mechanism of action. From this secondary screening we identified 21 compounds that showed an acetate-specific pattern of growth inhibition. These hits were further characterized for specific ICL- and MS-inhibition, and also for antibacterial spectrum of activity against other clinically-relevant Gram-negative pathogens.

### IC_50_ and MIC analysis of lead compounds demonstrates high affinity and broad spectrum activity against Gram-negative pathogens

In an attempt to confirm that the growth deficiencies seen in the primary and secondary assays were attributable to the specific inhibition of the glyoxylate shunt, the 21 compounds identified above were used to measure IC_50_ values against purified ICL and MS enzymes. The enzymatic assays used for each protein have been described previously [24], [25], and involve the detection of specific reaction products for each enzyme in the pathway. While all of these compounds had measurable IC_50_ values against at least one of the two enzymes, we found that 8 of them demonstrated inhibitory activities against both enzymes in the nanomolar to single-digit micromolar ranges (Table 1). Given that our *in vivo* results that suggested that dual-enzyme targeting would likely be required to eradicate an infection through glyoxylate shunt inhibition, together with the fact that resistance development is less likely to occur as rapidly when compounds have more than one target, we decided to further pursue only those compounds that showed inhibition against both ICL and MS. These 8 lead compounds were subjected to traditional MIC testing using both standard Mueller Hinton Broth (MHB) as well as the M9 Acetate medium that was used for primary screening. Interestingly, all 8 compounds demonstrated significant growth inhibition of 4 different Gram-negative pathogens when tested in M9 Acetate, yet showed little inhibition when these same strains were subjected to MIC testing in nutrient-replete MHB (Table 1). Given that most whole cell antibacterial screens are routinely conducted using a rich medium, it was not surprising to us that these compounds had never been identified from previous screening efforts. It is also worth noting that a decent correlation was seen between IC_50_ (against one or both enzymes) and MIC of many of the compounds tested (Table 1).

**Table 1:** MIC and IC_50_ values (in μg ml^−1^ and μM, respectively) for the 8 glyoxylate shunt-inhibiting compounds.

	MHB				M9 Acetate					
Compound	AB- 3167	EC 1545-08	KP MGH78578	PA PAO1	AB- 3167	EC 1545-08	KP MGH78578	PA PAO1	ICL IC_50_	MS IC_50_
1	>64	>64	>64	>64	2	16	8	8	0.69	0.18
2	64	>64	>64	64	2	2	2	4	0.24	1.77
3	32	32	64	32	4	4	4	8	0.17	5.30
4	64	64	64	64	1	1	1	2	0.03	1.29
5	32	32	64	64	8	4	4	16	0.37	2.35
6	64	64	64	64	4	4	4	8	0.09	2.73
7	32	32	64	64	8	4	4	8	0.19	6.93
8	64	64	64	64	8	4	8	16	0.37	8.88

### Analysis of compounds and path forward for medicinal chemistry lead optimization

**Figure 2:**
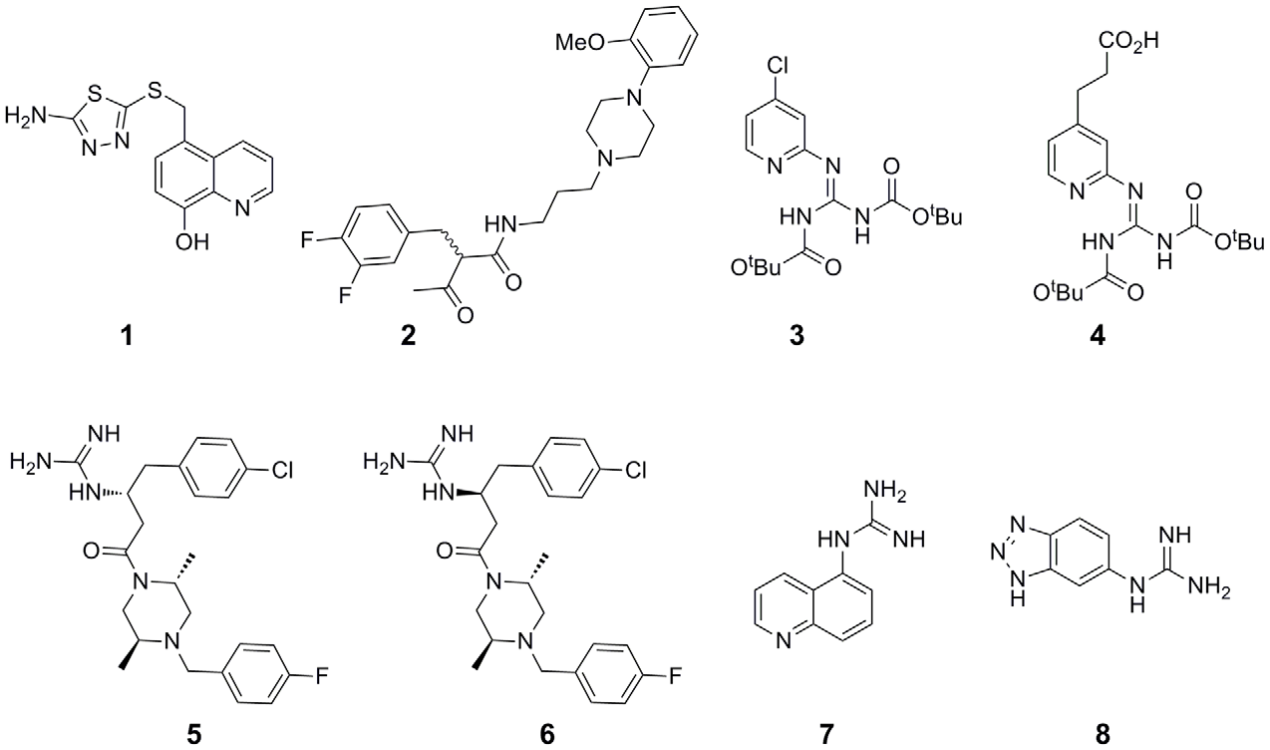
Chemical structures of the 8 *P. aeruginosa* glyoxylate shunt-inhibiting compounds.

The compounds identified in the HTS represent a variety of chemotypes; however, a number of common structure features exist between them, such as a piperazine and/or guanidine moiety flanked by a lipophilic, aromatic group (Figure 2). Within these hits there were sets of very closely related compounds. For example, compounds **3** and **4** differ by just one substituent on a pyridine ring; and piperazine compounds **5** and **6** are diastereomers of each other. Importantly, despite these structural similarities, there were measurable differences observed in the IC_50_s for these pairs against ICL or MS. Furthermore, these trends translated to the MICs determined in M9 acetate (Table 1). All of the hits identified are roughly rule-of-five compliant [39] with respect to molecular weight, lipophilicity (logP) and number of hydrogen bond donors and would represent reasonable starting points for future medicinal chemistry exploration.

**Figure 3:**
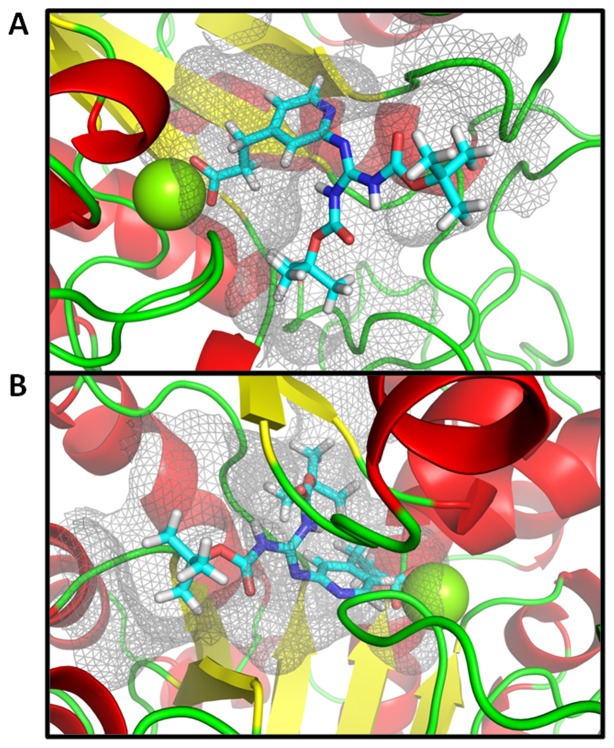
Structural modeling of Compound 4 bound to the *P. aeruginosa* glyoxylate shunt enzymes supports the dual-targeting capability of lead compounds. Compound **4**, docked with ICL (A) or MS (B), is depicted in a cyan-carbon colored stick representation, with the active sites of ICL and MS shown as mesh surfaces, the protein backbones in a ribbon diagram, and magnesium as a green sphere.

In order to develop avenues for optimization of these lead compounds and to convince ourselves that there would be a path forward for medicinal chemistry optimization via dual targeting of ICL and MS, we built homology models of *P. aeruginosa* ICL and MS using *M. tuberculosis* ICL [26] and MS [27] crystal structures as templates. We also developed an induced-fit docking model for all lead compounds. Figure 3 illustrates the binding mode of the most potent compound (Compound **4**) in both ICL and MS. Compound **4** binds in an extended conformation in both structures, indicating both that there is enough room for these compounds to bind and that further optimization by fine-tuning protein-ligand interactions to improve potency and other physicochemical properties is a viable path forward for a medicinal chemistry campaign.

## Conclusions

This work was conducted to explore the utility of alternative screening approaches for the identification of new antimicrobial lead chemical matter that had previously gone unidentified using traditional screening. By using a medium that exploits the functionality of an *in vivo*-essential pathway in *P. aeruginosa*, we found 8 different compounds (representing 5 different structural classes) that had impressive MICs against multiple clinically-relevant Gram-negative pathogens without any medicinal chemistry optimization. In addition, biochemical and structural modeling efforts suggest that these lead compounds are potentially binding to more than one target within the glyoxylate shunt pathway, a feature that has recently been emphasized to avoid rapid resistance development against newly designed and developed antibacterial agents [4]. While there is still much to be done to further characterize these compounds before they can be considered viable drug candidates, this study demonstrates that new leads can be identified from existing compound libraries, and that new pathways can be specifically targeted without the need for traditional target-based screens. This type of strategy should effectively complement efforts focused on natural product screening, fragment- and structure-based drug design, and other classical antibiotic drug development approaches to help generate new weapons in the war against MDR bacterial infections.
